# Demand-side financing in the form of baby packages in Northern Mozambique: Results from an observational study

**DOI:** 10.1371/journal.pone.0215282

**Published:** 2019-05-09

**Authors:** Anita Makins, Jochen Ehmer, Alexandra Piprek, Francisco Mbofana, Amanda Ross, Michael André Hobbins

**Affiliations:** 1 SolidarMed Mozambique, Pemba, Cabo Delgado, Mozambique; 2 SolidarMed Switzerland, Lucerne, Switzerland; 3 Ministerio da Saúde, Maputo, Mozambique; 4 Swiss Tropical and Public Health Institute, Basel, Switzerland; 5 University of Basel, Basel, Switzerland; Gettysburg College, UNITED STATES

## Abstract

**Background:**

The Maternal Mortality Ratio in Mozambique has stagnated at 405 deaths per 100,000 live births with virtually no progress over the last 15 years. Low Institutional Birth Rates (IBRs) levelling around 50% in many rural areas constitute one of the contributing reasons. Demand-side financing has successfully increased usage of maternal health services in other countries, but little information exists on in-kind incentives in rural Africa. The objective was to test the impact on Institutional Birth Rates of giving a USD 5.50 baby package incentive to every woman who came to give birth in a health centre in a rural, poor district of Cabo Delgado, Mozambique.

**Methods and findings:**

The intervention was implemented in one district in 2010 with the remaining 15 districts serving as controls. The total population in the 16 districts in 2006 was just under 1.5 million people. IBRs were observed from 2006 to 2013 (53 months before and 55 months after the intervention began). The non-intervention districts showed a slight increase, from a mean IBR of 0.39 (SD = 0.10) in 2006 to 0.67 (SD = 0.13) in 2014. The intervention district had a dramatic increase in IBRs within six months of the start of the intervention in 2010, which was sustained until the end of the study. Adjusting for the background increase and for confounders, including health facilities and health personnel per district, and taking clustering in districts into account, the estimated rate ratio of institutional births in the intervention district was 1.80 (95% CI 1.72, 1.89 p<0.001).

**Conclusion:**

Women were almost twice as likely to have an institutional birth following the introduction of the baby package.

## Introduction

Pregnancy and birth are critical periods in life. Globally, every year, one million babies die from complications during childbirth. An additional two million are stillborn due to problems arising during the last trimester of pregnancy [1]. Twenty million women suffer from pregnancy-related illnesses [2]. Every minute at least one woman dies from complications related to pregnancy or childbirth [3]. A large proportion of maternal and neonatal morbidity and mortality is preventable. Evidence-based, effective interventions to reduce pregnancy-related morbidity and mortality are well known and documented [4]. Resource-rich countries have implemented several interventions with success, and several resource-poor countries have undertaken fruitful efforts to scale them up, as was the case in Nepal [5]. Other countries have made less progress, especially in Sub-Saharan Africa [6,7]. As a result, more than 95% of maternal [8], neonatal [9] deaths and stillbirths [6] occur in low-income and middle-income countries. Inequalities also exist within countries; poor mothers in poor countries have less access to health care than wealthy mothers in poor countries [10]. Facility-based intrapartum care in the presence of a skilled birth attendant has been demonstrated to be a key strategy to reduce morbidity and mortality [10]. This paper focuses on increasing institutional birth rates as a strategy to improve maternal morbidity and mortality rates, although it is unable to document this as an outcome.

Mozambique currently ranks 181/188 on the UNDP human development index, with a GNI per capita of USD 1098 and a life expectancy of 55.5 years [11]. Seventy percent of the population live in multidimensional poverty [11]; 43% of the population have access to clean water and 19% to safe sanitation [12]. Sixty percent have no access to adequate healthcare and 68% of women are illiterate. Of the 28 million inhabitants, more than 2 million people are living with HIV/AIDS, affecting approximately 16% of pregnant women [13]. Maternal and neonatal health services are free of charge and, since the presidency of Samora Machel in 1975, are provided within a public system of health centres and hospitals.

Health service coverage, however, is low. Fifty-four percent of women give birth at home and only 2.2 health centres per 500,000 inhabitants provide Basic Emergency Obstetric and Neonatal Care (BEmONC) services as comparted to the WHO recommendations of 5 per 500,000, [14,15]. As a result, the Mozambican Maternal Mortality Rate remains high, with published numbers ranging between 408 and 600 [8,16,17]. Demographic and Health Services Surveys conducted in 2003 and 2011 detected no improvement. The lifetime risk for a woman dying from pregnancy-related causes is 1 in 42 [8]. And, although parameters in child health have improved, neonatal mortality rates continue high at 31 per 1,000 live births [2,17]. Hence, despite some variation in estimates, there has been little progress in improving maternal and neonatal health outcomes over the last 15 years.

Given the unpredictability of complications during birth, timely access to quality health services within the 24 hours surrounding birth is a critical factor in preventing maternal morbidity and mortality [4]. Thaddeus and Maine outlined the three barriers pregnant women face in reaching health care during labour: the decision to leave the homes to seek care; the ability to reach the facilities in a timely fashion; and the provision of quality maternal and neonatal services at the accessed health unit [18].

In impoverished settings in Sub-Saharan Africa, weak service provision is often seen as the main hurdle. This has been addressed by supply-side interventions such as improving midwifery training, health infrastructure expansion and extension of emergency obstetric services. These investments address the third delay and, although costly, tend to produce sustainable changes. However, in remote rural areas the first and second delay can sometimes be of more importance when it comes to saving women’s lives. The importance of the presence of the skilled birth attendant during child-birth is well accepted and in resource-poor countries this can only happen if the woman has an institutional birth. Consequently, the promotion of institutional births is recognised internationally as one of the core strategies for reducing maternal and neonatal deaths [4]. In a recent review of 80 lower and middle-income countries by Joseph *et al*. (2016), institutional births and births taking place with a skilled birth attendant were highly correlated, suggesting that most childbirth in the presence of a skilled birth attendant takes place at an institution. However, pro-urban and pro-rich inequalities were observed for coverage of institutional childbirth [10].

Subsequently, questions have arisen over how best to promote institutional births and overcome the barriers women may encounter in such poor settings. Various interventions have been designed to address particular barriers. For example, health promotion targets knowledge barriers, income generation targets economic barriers whilst motorbike ambulances target transport barriers. Another way of promoting behavioural changes and stimulating demand is through incentives. In the literature, the term used is ‘demand-side financing’ [19]. Incentives are a well-accepted method to promote changes in human behaviour, including in health [19]. In maternal health, there are examples in many countries, including India [20] and Pakistan [21], where they have yielded positive results. Various modalities such as conditional cash transfers, voucher schemes or in-kind incentives have been described in the literature. Conditional cash transfers have been used with some success in India and in South America, but with high transaction costs and inconsistent translation into improved health care outcomes [22,23]. These interventions have not been widely adopted in Africa. Voucher schemes can compensate providers for the reduction or abolition of user fees. There are examples in Uganda [24] where even private providers were included in such schemes. Evidence is still inconclusive with regard to demand-side financing, but its success appears to be context specific [25].

In-kind incentives are less well described in the literature. These do not reduce barriers but constitute tangible, immediate pull-factors encouraging pregnant women to overcome existing hurdles. A recent study in Zambia demonstrated a 43% increase in the institutional birth rate over one year following the introduction of an in-kind incentive that addressed a major barrier in this context: the demands made by health providers in health facilities for pregnant women to bring delivery supplies such as mother and baby clothes and delivery materials [26–28]. Apart from this, there is little evidence with regard to the effect of in-kind incentives in Sub-Saharan Africa. The aim of this study is to assess the impact on institutional birth rates of giving a baby package incentive to every woman who came to give birth in a health centre in a rural, poor district of Cabo Delgado, Mozambique. To our knowledge, this is the first documented study to examine the effect of an in-kind incentive such as a baby package as a form of demand-side financing in rural Mozambique.

## Methods

### Setting

Connected by one tarmac road to the rest of the country, Cabo Delgado is the most northern province of Mozambique. When compared to other provinces in the country, it has some of the poorest indicators as demonstrated in Table 1. In 2009, when the intervention was planned, Ancuabe had a population density of 22.6 people per square kilometre, the vast majority of whom lived off subsistence farming. Fifty-three percent of the population between the ages of 15 and 40 years of age were women. The population in the 16 districts were attended to by 110 health centres and 996 clinical staff.

**Table 1:** Health and context indicators in Cabo Delgado Province, by district.

	Neonatal Mortality Rate*	Perinatal Mortality Rate*	Infant Mortality Rate*	% pop with electri-city at home	% pop lowest 2 wealth quintiles	% women no schooling	% men no schooling	% pop using Family planning method
Niassa	28	31	61	90.8	37.6	45.6	30.5	12.4
**Cabo Delgado**	**31**	**62**	**82**	**5**	**61.5**	**49.4**	**36.3**	**2.9**
Nampula	15	24	41	85.5	51.8	37.1	21.4	5
Zambesia	37	30	95	93	69.7	34.8	16.7	4.7
Tete	48	54	86	88.2	46	41.2	29.2	15.3
Manica	23	30	64	77.8	18	22.7	10.5	13.2
Sofala	41	35	73	76.2	34.5	33.4	13.2	8.4
Inhambane	16	23	39	81.1	34.5	30.4	14.9	12.4
Gaza	34	43	63	76.5	16.6	28.9	19	18.3
Maputo Province	37	57	68	39.7	7.5	12.9	6.5	33.6
Maputo City	33	40	61	12.1	2.5	6.4	2.4	35.9
Mozambique	30	38	64	79.8	34.6	32.1	19.3	11.6

Data from DHS 2011 [16].

*per 1000 live births.

The intervention was performed in the district of Ancuabe where 112,610 (2009) people live mostly by subsistence farming. In the district, 99% of the population have no running water, 98% live in mud houses with straw roofs and only 56% have access to latrines (Table 2).

**Table 2:** Context indicators in the Ancuabe district compared to the remaining 15 districts of the province of Cabo Delgado, 2009.

	District Ancuabe	Remaining Districts of Cabo Delgado
Population	112 610	1 585 846
Nr of women between 15 and 45 years of age	24 481	350 996
Geographical area	4 984 km^2^	73 794 km^2^
Population density	22.6	21.5
Estimated births per year [based on 2007 census]	5068	71 363
Institutional Birth Rate	54.1%	58.90%
% Caesarean Section	1.46%	1.25%
Nr of health centres	6	104
Nr of CEMONC centres	0	4
Nr medical technical staff	36	960

Data according to the National Health Information System (NHIS). CEmONC: Comprehensive Emergency Obstetric and Neonatal Care.

There was no facility providing Comprehensive Emergency Obstetric and Neonatal Care (CEmONC) in the district. On average, women travelled 10 kilometres to reach the nearest health centre. The remaining districts of the province served as control districts.

### Study population and observation time

The study was carried out in all 16 rural districts of Cabo Delgado Province, comprising one intervention district (Ancuabe) and 15 comparison districts. The distribution of baby packages was implemented in Ancuabe beginning on June 1, 2010. For all 16 Districts, the number of institutional births was observed from January 2006 to December 2013, comprising 53 months before and 55 months after the start of the intervention.

At the start (2006), the 16 Districts had a total population of 1,433,344, 7% of whom (104,694) lived in the intervention district of Ancuabe, and 83% (1,328,650) in the 15 non-intervention rural districts. The population of the province reached a total of 1,671,344 by 2013. Due to its different social, geographic and service coverage pattern, the only urban district of the Province, the capital Pemba (which had the provincial referral hospital) was not included in the analysis. Therefore, its population of 135,438 was excluded, making the control and intervention districts more comparable.

### The intervention

The intervention consisted of giving a ‘baby package’ to every woman who had a birth (live or stillbirth) in one of the 6 health centres in Ancuabe district. This is in line with national policy guidance that identifies incentives as one of the ratified strategies to increase institutional birth rates. Those who had twins received two baby packages. The baby package consisted of a traditional cloth (*capulana*), a plastic basin, a bar of soap and 3 cloth nappies. The choice of items was decided following focus group discussions with women’s groups before the start of the intervention. The package was the same for all women, independent of income or distance from the health centre. The mean cost per package was USD 5.50, including transport of the materials. No promotional campaign was organised. Packages were bought from a bulk supplier in the city of Nampula on a six-month basis. The supplier organised the 250km transport to the district store room. The Chief District Health Nurse then distributed the packages on a monthly basis from the central district store to each health centre, with occasional transport support from the Swiss non-profit organization, SolidarMed. The package was given to the mother by the nurse at each health centre upon discharge and her name and fingerprint were recorded as confirmation of receipt. Every three months, stocks were monitored by a nurse who reconciled the number of distributed packages with the number of institutional births occurring in each health centre, in order to avoid fraud. Health centre stock-out of baby packages occurred on only one occasion (February 2012), due to delays in purchase and delivery.

No supply side interventions were implemented concurrently with baby package intervention over the time period monitored.

### Data collection

For the purpose of stock control, a paper-based register was introduced at all participating health facilities in the intervention district. For each baby package given, the nurse from the health centre was asked to record the name and age of the mother, the date of delivery, mother’s village of origin and distance from the health centre. Once a month, the study nurse entered those data into a spreadsheet by Microsoft Excel (version 2010) and gave stock monitoring feedback to the nurse at each health centre. Monitoring visits were performed every 4 to 6 weeks by NGO staff to ensure that the baby package register was correctly filled and that patients were receiving the baby package as registered.

Institutional birth numbers used for this study were not, however, extracted from the baby package register, but from the Mozambican National Health Information System (NHIS), which was in place prior to the baby package intervention and was not modified for the study purposes.

To assess the accuracy of the NHIS data, we compared the number of institutional births in the intervention district as registered by NHIS with the numbers of institutional births registered by the baby package registers. The discrepancies in the year 2010, 2011, 2012 and 2013 were 2.2%, -1.1%, 0%, and 0.003%, respectively. This indicates an overall good quality of NHIS data. The NHIS is routinely checked by the government HIS department. No other additional data checks were implemented for the purposes of this project.

The number of institutional deliveries which occurred in each rural district was extracted from the NHIS, which defines institutional delivery as “giving birth in a public health facility with assistance from a trained provider”[14].

To calculate institutional delivery rates, we divided the numbers of institutional deliveries by the number of expected deliveries in each district. The Ministry of Health calculates this denominator by using the same yearly crude birth rate of 45 per 1000 which is multiplied by the estimated population for that year. These population estimates in turn are based on yearly projections based on the 2007 census published by the Instituto Nacional de Estatistica which are used by the National Health Information System. Given inaccuracies in these estimates, the number of recorded deliveries in the intervention district turned out to be greater than the number of expected deliveries, resulting in coverage of more than 100%.

The denominator of expected deliveries does, however, provide a basis on which to measure the rates of institutional births, as there is no obvious reason why the error would differ between districts and therefore is not expected to introduce bias in the estimate for the effect of the intervention. In our analysis, we used the rate of institutional deliveries, rather than the proportion, since a proportion cannot be greater than 100%.

The baby package intervention commenced on 1^st^ June 2010. In order to divide the year into before and after the start of the intervention, the number of institutional births in 2010 was divided into January to May and June to December. The expected number of births for the two parts of the year was calculated assuming that there was no seasonality in the number of expected deliveries.

### Statistical analysis

Since the outcome of institutional delivery is a rate, we used Poisson regression to estimate the effect of the baby package on the number of institutional deliveries per expected birth. We checked the assumptions and the fit using residual plots. We allow for the background increase in institutional delivery rates in the 15 rural control districts. After graphical checks, we assumed a linear increase over time. To account for the clustering within districts, we include random effects for the districts for the intercept and slope which allows both the absolute starting value of institutional delivery rate and the background increase to vary by district. We adjusted for the potential confounders, the number of health facilities per district and the number of health personnel per district. We then estimated the additional increase associated with the intervention. Following graphical checks, we find that there is a sudden effect of the intervention and that this increase is maintained over time.

### Ethical considerations

The study is a retrospective analysis of data collected by the routine health registration system which consists of aggregated data over time. The implementation of the baby package incentive followed national recommendations and was distributed by the Ministry of Health. As such, it was part of a routine health service strategy implementation. No personal data were collected and/or included for the analysis. Ethical clearance was sought from the head of the ethics committee at the time of implementation. It was confirmed that no ethical approval nor consent was necessary for the analysis given the routine nature of the proceedings. Baby packages continue to be offered routinely to all women who give birth in a health centre in the district of Ancuabe as of the date of submission of this manuscript. The findings reported in this paper have been disseminated at provincial and national level on multiple occasions, in both scientific forums and Ministry of Health meetings.

### Conflicts of interest

The study has been designed and implemented by the Swiss Non-Profit Organization SolidarMed in conjunction with the Mozambican Ministry of Health. SolidarMed has been working in Mozambique for 20 years collaborating with the Ministry of Health in order to improve health care in the province of Cabo Delgado. The baby package intervention was designed and implemented by SolidarMed and the Ministry of Health in partnership. There were no other partners. Data were not collected specifically for the intervention. All data analyzed were routinely collected across all districts through the Ministry’s routine health management information system and therefore could not be tampered with or altered in any way. There were no incentives or benefits to any partner, implementing health worker or individual health centre, which was linked to the success or failure of the intervention in any way. There is no known conflict of interest to be declared.

## Results

**Fig 1:**
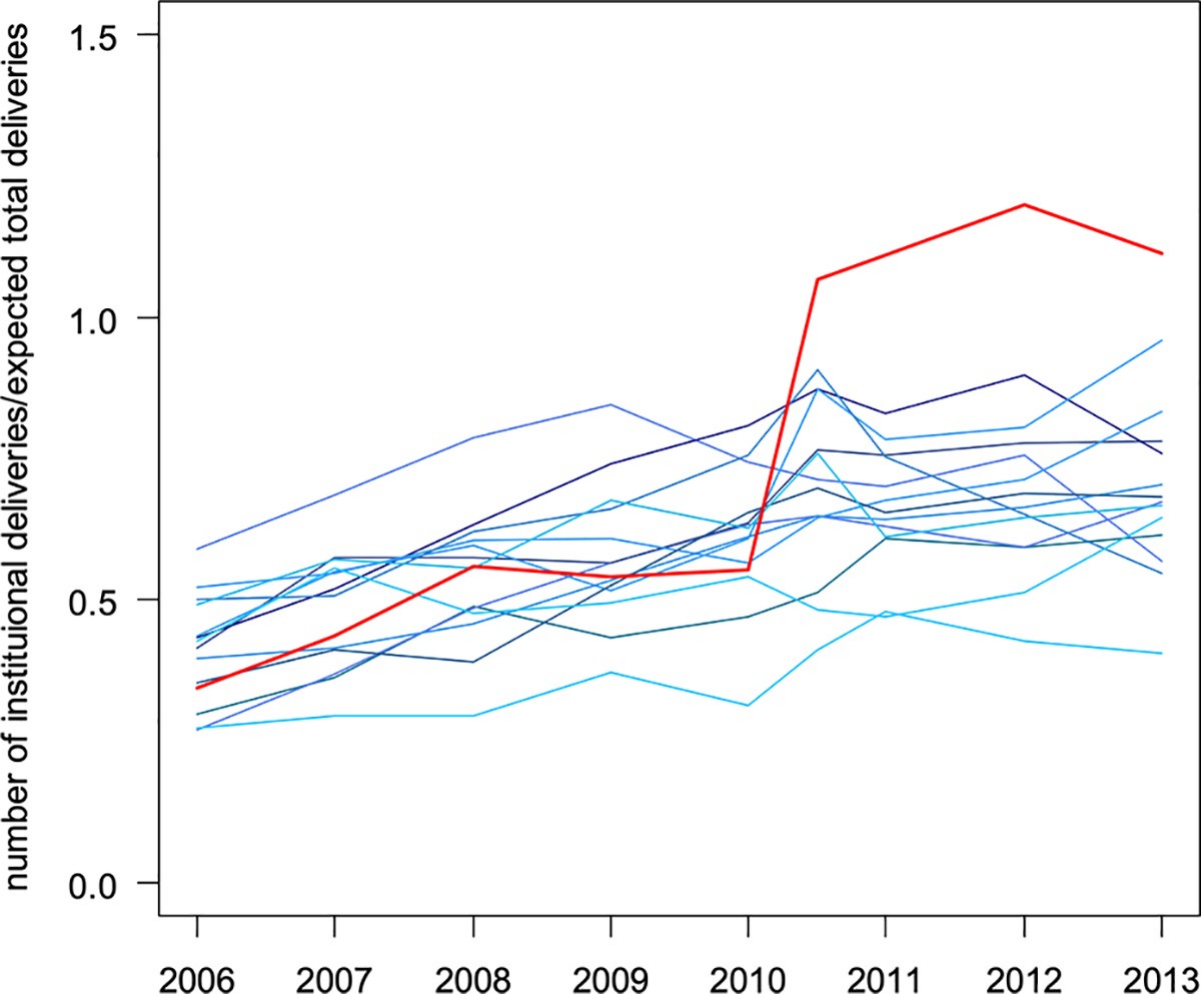
The rate of institutional deliveries per estimated delivery for the individual districts over time. The red line represents the intervention district Ancuabe. The blue lines represent the districts without the baby package intervention (Balama, Chiure, Ibo, Macomia, Mecufi, Meluco, Mocimboa da Praia, Montepuez, Mueda, Muidumbe, Namuno, Nangade, Palma, Pemba Metuge, Quissanga).

In the observation period comprising 53 months before and 55 months after the start of the intervention, 634,529 births were expected to take place in the 16 study districts (7.3% in the intervention district and 92.7% in the comparison districts). These include both home and institutional births. A total of 375,850 institutional deliveries were recorded. In 2006, the institutional delivery rate in the intervention district of Ancuabe was 0.34 institutional births per expected birth compared to 0.39 (SD = 0.10) in the 15 rural non-intervention districts. Over time, the institutional delivery rates for the non-intervention districts show a moderate increase from the mean rate of 0.39 to 0.67 (SD = 0.13), with variation among the districts (Fig 1 and S1 Table).

In contrast, with the introduction of the baby package intervention in June 2010, there is a sudden increase of the institutional delivery rate in the intervention district. This increase was sustained until the end of the observation period, albeit with fluctuations. It is the intention of the authors to continue observations once the intervention ceases in order to assess sustainability of the effect in the future.

Adjusted for the background increase in the non-intervention districts as well as for the number of health facilities and technical health personnel per district, the effect of the intervention was estimated to be a rate ratio of 1.80 (95% CI 1.72, 1.89 p<0.001). This suggests that women were almost twice as likely to have an institutional birth following the introduction of the baby package. An increase in the influx of women from neighboring districts into the intervention district was observed, which plateaued at around 180 per year by 2013. However, this represents only 3% of the number of institutional deliveries in the intervention district in the same year.

## Discussion

This study suggests that demand-side financing in form of a USD 5.50 baby package was a sufficient pull factor in the rural district of Ancuabe to double institutional birth rates and sustain them at that level. The importance of this finding lies in the known clinical benefits of an institutional birth compared to a home birth in the rural African context. In low resource settings, home birth is a hazardous affair both for the mother and the neonate. The importance of access to health services during the ‘golden 24 hours’ surrounding birth are well understood [4] as are the health benefits of avoiding unhygienic birth conditions and poor practices common in rural home births. The baby package in rural Ancuabe seemed to resolve steps 1 and 2 in Thaddeus and Maine’s description of the barriers faced by pregnant women in accessing appropriate health care [18]. In addition, institutional birth links women to other beneficial services such as breast-feeding counselling, family planning, prevention of vertical HIV transmission, timely neonatal care in the event of complications and vaccination. Institutional births are thought to reduce neonatal mortality by 29% [29]. The results are clear-cut and are also consistent with previous findings in Zambia [27].

The intervention had multiple strengths. First, in the Mozambican context of absolute poverty, the content of this simple USD 5.50 baby package is clearly highly valued by the mother, possibly because it supports her in looking after her newborn baby. The provision of an in-kind incentive allows for a combination of both intrinsic and extrinsic value. The continuous availability of the baby package at district health centres, without stock-outs of any considerable duration, is likely to have strengthened this value. While not included in Mozambique’s strategy, monetary incentives might have achieved a similar effect, but could also have entailed a greater risk of fraud or expropriation by the partner or wider family, and thus raise ethical questions [30]. In a qualitative evaluation in India, the effect of the cash transfer program was confirmed, but it seemed to have had a lesser role in the overall impact compared to other enabling factors such as the arrangement of transport, escort to and support at the health care facility [31]. Voucher models would have made sense to reduce or abolish user fees, but not in a context of already free services. Furthermore, voucher models involve considerable investment in management, control and reimbursement systems, and take away the immediateness of the reward. Ongoing research by Dumbaugh *et al*. on the comparative effectiveness of different demand-side interventions to increase utilization of maternal health services in the Democratic Republic of the Congo promises to shed more light on how the individual interventions may impact populations and outcomes at different levels [32].

A second and important strength of the study lies with its implications for equity. Despite some progress in some Lower and Middle Income Countries, inequity and inequality in coverage remain a serious challenge [10]. To achieve 100% institutional birth coverage, a pull factor needs to be of sufficient power to deploy its effect over different socioeconomic and distance strata, without leaving specific sub-groups behind. This seemed to be the case in Ancuabe. However, given the slight inaccuracies regarding population estimates, it would be prudent to engage in further qualitative research to investigate how factors such as socioeconomic status, distance, educational level and comorbidities (e.g. HIV) may have influenced the subjective appreciation of the incentive and hence understand better the determinants of value-appreciation.

Our study comes with limitations. First, due to resource constraints, we did not design it as a cluster randomized control study but chose an observational study design. This mirrored best a natural experiment of the implementation of a national strategy. Furthermore, randomization of the intervention within one district–e.g. by health centres as clusters–would have been highly prone to contamination (women deciding to give birth at a centre with demand-side financing scheme, despite greater distance) and randomization among districts would have surpassed our implementation capacity. The observational study design comes with known limitations of bias and confounding, for which we accounted as far as possible at the analysis stage. Second, the study depended on census data for the number of expected births, and therefore the calculation of the rate instead of proportions. Third–as a proof-of-concept study–the results do not inform about aspects of feasibility and implementation under routine Ministry of Health stewardship. The generalizability of its results may be questionable when considering contexts different from the one described in Ancuabe, a homogenously poor rural district. The importance of context specificity in overcoming barriers to access to care was highlighted in a recent systematic review by Kyei-Nimakoh *et al*. [33]. In other words, one should take caution when considering implementation in urban districts, those with greater variation in socioeconomic status and those with vastly differing geographical realities (i.e. distance and lack of transport to health centres are likely to affect outcomes). It would also be informative to assess the sustainability of the effect, once the intervention is discontinued. Fourth, although the desirable ultimate effect of the baby package is to improve maternal and neonatal morbidity and mortality, measuring those outcomes was beyond the scope of this study. The study relied on the available aggregated indicator of the “number of institutional births” at the NHIS, which showed very high accuracy when compared to a separate baby package stock register. Institutional births in such settings were also shown to be associated with childbirth with a skilled birth attendant [10], which is commonly understood as a pre-requisite for the reduction of maternal and neonatal mortality. While this intervention tackles the delays of seeking and reaching care, it does not evaluate the adequacy of care received at the facility. In a study context such as the one described–low resource setting with poor infrastructure, staffing levels, medicines, equipment, technology and service coverage–the need for additional supply-side interventions is obvious. In our experience, the increase in the number of institutional births did indeed put additional pressure on existing infrastructure, human resources and medical equipment. But it also triggered efforts by the Ministry of Health to provide staff and equipment in those health centres where resources were insufficient. In general, nurses coped well with the demand, and job satisfaction in many instances was augmented with the realization that their expertise and services were valued and sought after by the population. In a second phase of the intervention, improving the quality of care is proceeding and further research will allow for a better understanding of the effect of complementary supply-side interventions.

The study was not designed to inform about cost. effectiveness. Although the unit price of USD 5.50 including logistics is modest, the number of DALYs averted per USD would also be driven by the mortality and morbidity outcomes. Preliminary results on a subsequent comparative cost analysis in the same region presented by Hobbins, suggest that demand-side and supply-side interventions may yield the greatest cost effectiveness when implemented together [34]. Although there are other examples of successful demand-side financing in maternal health [20,21,27], this is one of the first studies to analyze an in-kind baby package incentive in a rural, very poor area of Sub-Saharan Africa. It demonstrates that a relatively simple intervention can have a dramatic impact in a short period of time.

It is likely that if complemented by efforts of strengthening the quality of obstetric services, this in-kind demand-side intervention has the potential to accelerate progress towards improved maternal and neonatal health outcomes in resource-poor settings rapidly [4,29].

Although simplicity, robustness and the need for few extra logistical and managerial resources were purposefully considered in the design of this study, a larger-scale proof-of-implementation study is recommended to inform public health policy and to roll this intervention out confidently at the national level.

## Supporting information

S1 Table Key Indicators for all districts of the province of Cabo Delgado, 2006–2014. (XLSX)

S2 Table Key confounders for all districts of the province of Cabo Delgado, 2006–2014. (XLSX)
